# Hiding the squid: patterns in artificial cephalopod skin

**DOI:** 10.1098/rsif.2015.0281

**Published:** 2015-07-06

**Authors:** Aaron Fishman, Jonathan Rossiter, Martin Homer

**Affiliations:** Department of Engineering Mathematics, University of Bristol, Bristol BS8 1UB, UK

**Keywords:** artificial chromatophores, dynamic pattern generation, artificial skin, biomimetic, dielectric elastomer

## Abstract

Cephalopods employ their chromomorphic skins for rapid and versatile active camouflage and signalling effects. This is achieved using dense networks of pigmented, muscle-driven *chromatophore* cells which are neurally stimulated to actuate and affect local skin colouring. This allows cephalopods to adopt numerous dynamic and complex skin patterns, most commonly used to blend into the environment or to communicate with other animals. Our ultimate goal is to create an artificial skin that can mimic such pattern generation techniques, and that could produce a host of novel and compliant devices such as cloaking suits and dynamic illuminated clothing. This paper presents the design, mathematical modelling and analysis of a dynamic biomimetic pattern generation system using bioinspired artificial chromatophores. The artificial skin is made from electroactive *dielectric elastomer*: a soft, planar-actuating smart material that we show can be effective at mimicking the actuation of biological chromatophores. The proposed system achieves dynamic pattern generation by imposing simple local rules into the artificial chromatophore cells so that they can sense their surroundings in order to manipulate their actuation. By modelling sets of artificial chromatophores in linear arrays of cells, we explore the capability of the system to generate a variety of dynamic pattern types. We show that it is possible to mimic patterning seen in cephalopods, such as the passing cloud display, and other complex dynamic patterning.

## 1. Introduction

**Figure 1.**
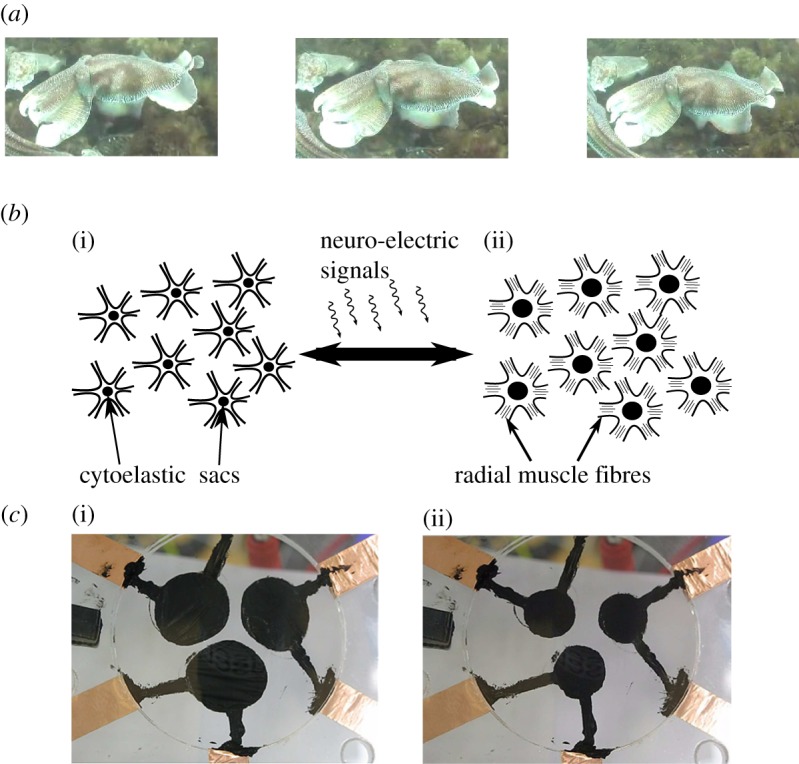
(*a*) The passing cloud display of the *Sepia apama*. (Reproduced under a Creative Commons license (CC BY-NC-SA 3.0) [2].) (*b*) Illustration of cephalopod chromatophores in unactuated (i) and actuated (ii) states driven by the activation of muscle fibres which expand and contract pigmented cytoelastic sacs. (*c*) Three prototype artificial chromatophores are shown in unactuated (i) and actuated (ii) states and are made from DE using 3M VHB4905 tape coated with black carbon grease electrodes [3]. (Online version in colour.)

In this paper, we present an application of smart materials, inspired by biological chromatophores, to generate active dynamic patterns. Chromatophores are small pigment-containing cells embedded into the skin of animals, such as amphibians, reptiles and fish. In particular, the cephalopod employs its skin for controlled chromomorphic effects, serving as effective tools for signalling and camouflage [1]. For example, the *Sepia apama* is known for its ‘passing cloud’ display, where bands of blue-green colour propagate as waves across the skin, as shown in figure 1*a*. This visual effect acts to distract and divert predators. Individual chromatophores actuate under neuro-electrical stimulus of flat wedge-shaped muscles [4], causing the pigmented region (saccule) to increase in area and affect local skin colouring, as illustrated in figure 1*b*. The tight, neuro-muscular connections coordinate actuation, allowing cephalopods to rapidly shift between skin colours and pattern types.

Biomimetic cephalopod chromomorphism allows for new approaches to camouflage and signalling using soft and compliant artificial skin. Compared with other proposed active camouflage solutions, such as retroreflective projection technology [5], an artificial skin requires no external projectors, making it easier to maintain patterns when mobile. By employing complex and dynamic patterns, users may stand out in times of danger, useful for signalling applications such as search and rescue operations.

One promising class of materials for creating artificial muscle is electroactive polymers (EAPs), smart polymers that exhibit a change in shape or size in response to electrical stimulus. Their properties are well suited to mimicking muscular systems in animals since they can be engineered into lightweight and compliant actuators with relatively high work output [6]. Many biological functions have been successfully mimicked using EAPs, including artificial cilia using ionic polymer–metal composites (IPMCs) [7] and the swimming motion of eels using polymer gels [8].

Dielectric elastomers (DEs), a type of planar-actuating EAP, offer potential towards realizing effective artificial cephalopod skin. Previous studies investigating a three-spot artificial DE chromatophore system (shown in figure 1*c*) indicate significant optical modulation that is of the same order as cephalopod chromatophores [3]. Furthermore, their compliance and rapid actuation speed should allow for wearable biomimetic technologies which respond quickly to stimuli.

For these reasons, this study focuses on using DEs for pattern generation in artificial cephalopod skins, and describes how they can be designed, mathematically modelled and analysed. We develop a hyperelastic electro-mechanical model and use it to simulate a variety of dynamic patterns using artificial chromatophores, composed of a linear array (thread) of DE. The DE thread is divided into cells, each of which is capable of sensing local or remote strain through strain-response switches, such as the DE switches developed by O'Brien *et al.* [9]. These sensors can be considered analogous to the strain-sensing spindle cells found in biological muscles [4]. By imposing simple rules that dictate the local interaction of neighbouring artificial chromatophores, we generate a range of complex and controllable patterns such as unidirectional propagation and sawtooth-like oscillation. We demonstrate that the generalized system design allows mimicry of dynamic skin patterning seen in biological chromatophore systems including the passing cloud display and other complex behaviour.

This paper continues by outlining a generalized pattern generation system made from artificial chromatophore cells in §2. Section 3 then integrates this system into a time-dependent hyperelastic model that captures the physical and mechanical properties of DEs. This section also presents a discretization scheme of the model into linear arrays of artificial chromatophores or *threads*. Section 4 proceeds with the implementation of rule bases, using the discretization scheme, to generate a selection of biomimetic patterns including sawtooth oscillation, unidirectional propagation and complex behaviour. The paper concludes with a discussion of the potential of our system for future pattern generation in artificial skin.

## 2. Artificial chromatophore system design

The proposed artificial chromatophore system consists of a sheet of DE with a number of coloured electrodes painted on the material, forming a set of actuators or artificial chromatophore *cells*. The aim of our approach is to examine how patterns propagate within a chromatophore skin-like material, without the complexity of top-down independent control of each cell. We implement two types of cell, which differ in the control of their actuation. To reflect the intrinsic behaviour of chromatophores, *self-sensing* cells use control that is dependent on deformations within the material. Extrinsic behaviour is included using *manual* cells, which may control their actuation according to any external stimuli in the material environment (e.g. light or heat), or, indeed, external neural input.

**Figure 2.**
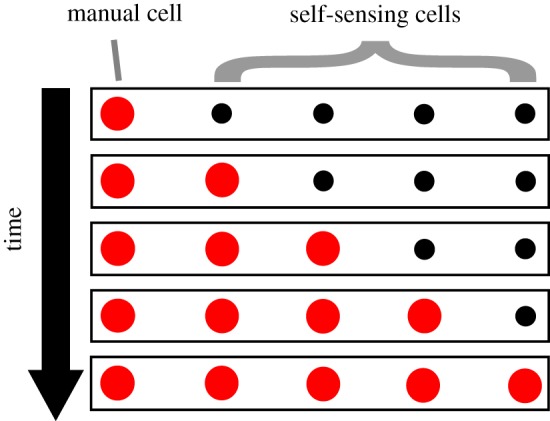
A conceptual example implementation of manual and self-sensing cells in a linear array, constrained between fixed end boundaries, with the switch of the manual cell closed. Small spots indicate the cell is not actuated and large spots indicate the cell is actuated. (Online version in colour.)

As an illustrative example, consider a manual cell followed by a series of self-sensing cells as shown schematically in figure 2. If the manual cell is stimulated the deformation would be detected by a neighbouring self-sensing cell, prompting it to actuate, given the right control rule. This could potentially cause a ‘chain-reaction’ of actuation along the series of self-sensing cells, where the final state of the system has every cell actuated. Similarly, the contraction of the manual cell could also cause contraction propagations that cause the chromatophore system to approach its original state. This simple example illustrates a mechanism of transitioning between two pattern types: ‘all unactuated’ to ‘all actuated’.

We now assume that each cell is charged using an individual RC (resistor–capacitor) circuit connected in series to a source voltage. This models the finite resistance of the conducting electrodes of the DE actuator, coupled to its capacitance, as it charges and discharges during actuation. We equip each circuit with a binary switch, which immediately begins to charge the cell when closed. Self-sensing and manual cells differ in the control of this switch.

Self-sensing cells can be thought of as agents that decide when to contract and expand by choosing when to open and close their switch. To make such decisions the agents have two (local) pieces of information available to them: (i) the current state of their switch and (ii) their *source*, a scalar function of the current deformation. For example, the source may be the mean strain of the agent's cell, or the mean strain of its neighbouring cells. The agent closes its switch when the source lies within pre-defined ranges, forming an *activation rule* for the cell to expand. If the switch is closed and the source lies within a second set of ranges, the agent will open its switch, contracting the cell and forming the *deactivation rule*. Both rules together form the *rule base* of the system.

In practice, rule bases could be implemented using the dielectric elastomer switches (DES) proposed in [9]. By embedding DES close to a cell, we can use changes in resistivity to infer the cell's source and control its actuation through digital circuits. That being noted, an implementation of such a system is outside the scope of this paper, and for simplicity we will assume that perfect knowledge of the cell's strain is available for use in control.

The manual cells have much flexibility in their implementation and their control. In this paper, we use their actuation as triggers that prompt self-sensing cell switching to change the material pattern type. The cause of their actuation may be controlled by, for example, a toggle switch, or a photodiode in conjunction with a voltage gate.

## 3. Mathematical model

This section describes the operation of DE actuators and framework required to mathematically model the proposed DE pattern generation system. As introducing sets of switches increases system complexity, we discuss how we can characterize the state of a system using both the states of the switches and the motion of the material. We then introduce our viscoelastic continuum model, developed specifically for these DEs. This section concludes with a discussion of the model parameters fitted for 3M's VHB4905 [10], a high-performance, and commonly used, DE that we consider throughout the rest of the paper.

### 3.1. Dielectric elastomers as artificial muscles

**Figure 3.**
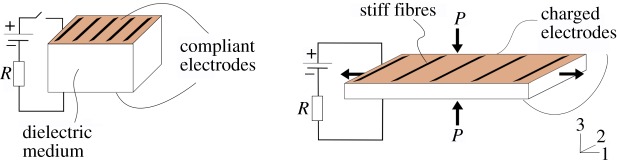
Schematic of the actuation of uniaxial DEs. Applying a potential difference across the dielectric medium causes charge to build on the electrodes. The resulting Maxwell stress on the plates, *P*, caused by Coulomb attraction between the opposing charges on the plates, creates an expansion normal to the plane of the electrodes. The incompressibility of the elastomer forces the DE film to contract in the thickness direction 3 and expand in the longitudinal direction 1. Deformation in direction 2 is prevented by stiff fibres embedded onto the surface of the membrane. (Online version in colour.)

When coated with a compliant electrode, sheets of DE store charge, much like a parallel plate capacitor. The build-up of charge induces Maxwell stresses within the material. For incompressible elastomers, this causes the material to expand in the plane of the electrodes and contract in the direction normal to the electrodes. For simplicity, we consider a situation in which the DE is constrained in one direction by light, stiff, parallel fibres added to the surface of the membrane in the plane of the electrodes [11]. The effect of the fibre constraint is to fix the length of the membrane in the direction of the fibres, so the expansion of the DE is uniaxial, normal to the fibre constraints, as illustrated in figure 3. It is known that a membrane subjected to uniaxial force along its length can achieve large deformation when the width direction is constrained [12]. Material undergoing such electrostatic expansion is defined as *active* and is *passive* otherwise. It has been shown [13] that the Maxwell stress, *P*, relates to the thickness of the elastomer, *d*, with applied voltage, *V*, by3.1
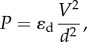
where *ɛ*_d_ = *ɛ*_r_*ɛ*_0_, *ɛ*_0_ is the permittivity of free space (approx. 8.85 × 10^−12^ Fm^−1^) and *ɛ*_r_ is the relative permittivity of the dielectric (typically around 4). We define active material that has approached steady state to be *actuated* and is *unactuated* otherwise.

### 3.2. Global and local dynamics

Including switches and sensors in the DE means that the motion of the material may be described by a dynamical system that is piecewise smooth [14]. Discontinuities occur at the crossing of thresholds, where the closing and opening of switches result in fast changes in system variables such as applied stress. To assess such discontinuities, we introduce the *global state* of the system, defined as the set of on/off states of all the switches. The system transitions between these discrete states when the material crosses a threshold.

We define *g_i_* to be the state of switch *i* with *g*_*i*_ ∈ {0, 1}, where 0 denotes the switch is open and 1 denotes the switch is closed. The global state of the system with *n* switches, one per cell, *G* is then defined to be *G* = [*g*_1_, *g*_2_, …, *g_n_*]. The global state does not uniquely define the material configuration, but is instead used to determine the electro-mechanical dynamics throughout the material.

The transition to the next global state, if any, is determined by the dynamics that occur when in a global state, described by a smooth dynamical system unique to that global state. We refer to this as the *local dynamics*. The initial conditions and governing dynamics of the global state determine which global state the system will transition to, as well as providing the initial conditions for the next global state.

### 3.3. Viscoelastic model

Our proposed viscoelastic model is based on that in [15] and has been chosen for its tractability and consistency with experimental data at high strains, for both fast and slow strain rates. Other viscoelastic models, such as in [16], are described using delay-differential equations (DDEs) and, although accurate, require sophisticated numerical integration schemes as well as being computationally expensive. Simpler models, such as [17], incorporate hyperelasticity into a Maxwell viscoelastic model [18]. Although these models are more tractable, studies indicated they were inaccurate for stretch ratios larger than 3 [19].

The DE is modelled using the hyperelastic Gent strain energy form [20]. The strain energy function, *W*, has the general form
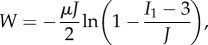
where *μ* and *J* are material constants and *I*_1_ is the first invariant of the deformation gradient written in terms of principal stretches in coordinate direction *k*, *λ*_*k*_, as 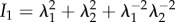 for an incompressible material. Because the DE we consider in this paper is fibre constrained in the width direction, 2, the principal stretch *λ*_2_ is a constant that we can choose (within limits), the prestretch *λ*_2,pre_. Hence we need only determine the behaviour of the material in the longitudinal, 1, direction. In practice, to prevent buckling and improve actuator performance, the DE is also prestretched in the longitudinal direction [21].

The Cauchy stress in the longitudinal direction, 1, of a generic hyperelastic element is given by3.2
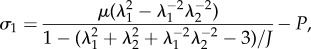
where *P* is the Maxwell stress.

**Figure 4.**
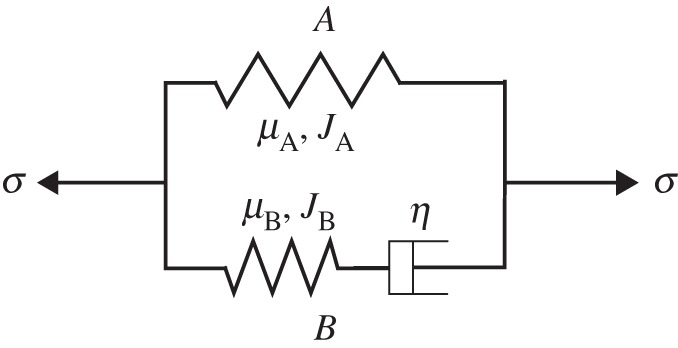
Rheology of the viscoelastic model.

We model the continuum as two parallel superposed networks, as in [15]. The first network, *A*, consists of a hyperelastic spring, while the second, *B*, consists of a viscous dashpot and a hyperelastic spring connected in series. Such rheology, summarized in figure 4, has been shown to successfully model time-dependent behaviour for DEs in numerous applications [22,23]. Both hyperelastic springs are modelled using the Gent strain energy form [20].

Following this rheology, we let *ξ*_*k*_ and 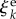 represent the principal stretches in direction *k* over the viscous and elastic components, respectively, of network *B*, and *λ*_*k*_ the corresponding total stretch in the network (note, again, that the stretches in direction 2 are constant in our implementation). Using multiplicative decomposition, the elastic stretch is given by 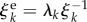 [23], meaning that larger viscous stretches result in lower elastic stretches.

The total stress is found by summing the contribution from the elastic components in each network. Let *μ*_A_, *μ*_B_, *J*_A_ and *J*_B_ represent the material constants in the hyperelastic components of networks *A* and *B*, respectively. Using (3.2), the Cauchy stress in direction 1 of the material is3.3
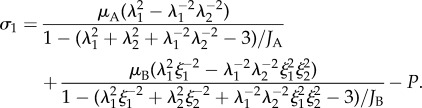


The first and second terms of (3.3) represent the stresses from network *A* and *B*, respectively. From (3.3) we note that, in general, larger viscous strains result in lower Cauchy stresses. Therefore, larger differences between *λ*_*k*_ and *ξ*_*k*_, which can arise from fast straining in the material, often result in greater resistances to motion.

The rate of change of the viscous component in network *B* is found by modelling the dashpot in network *B* as a Newtonian fluid [15], yielding3.4
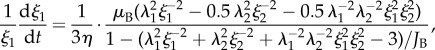
where *η* is the viscosity of the dashpot. For larger *η*, the rate of plastic deformation is slower, resulting in a longer relaxation time. The viscosity and spring modulus define the relaxation time, *τ* = *η*/*μ*_B_ [15].

In order to model the longitudinal prestretch, we consider the material to have been fixed to the desired ratio in the 1-direction, *λ*_1,pre_, before waiting a long time for the stresses in the viscous component to approach steady state. At steady state, we have *ξ*_1_ = 0, which implies that *λ*_1_ = *ξ*_1_. The initial conditions for a prestretched material starting from rest are then defined as *λ*_1_|*_t_*_=0_ = *ξ*_1_|*_t_*_=0_ = *λ*_1,pre_. Similarly, the fibre constraints imply that *λ*_2_ = *ξ*_2_ = *λ*_2,pre_ for all *t*.

Time-dependent behaviour is modelled using the constitutive equations (3.3) and (3.4) and requires the parameters *μ*_A_, *μ*_B_, *J*_A_, *J*_A_ and *τ*. We are particularly interested in using our model to assess VHB4905, which has been shown to be capable of exhibiting linear actuation strains of over 500% [24], in the same order of magnitude as cephalopods' chromatophores [3]. Consistent with experimentation, this study uses proposed limits of material strength failure occurring when *λ* = 6 [10]. VHB4905 is manufactured with a thickness of 0.5 mm, which is a convenient thickness for designing artificial cephalopod skin. Since VHB4905 has a Poisson ratio of 0.49 [10], this study assumes it is fully incompressible. The material model parameters, given in table 1, have been fitted to existing experimental data [23].

**Table 1.** Material parameters used in the model.

parameter	value (unit)
*μ*_A_	25 000 (Pa)
*μ*_B_	70 000 (Pa)
*J*_A_	90
*J*_B_	30
*τ*	3 (s)
*ɛ*_d_	4.5

### 3.4. Discretization of the viscoelastic model

In order to simulate and analyse our model, we discretize the material geometry. For simplicity, we consider the unidirectional motion of a membrane that is fibre constrained in the width direction. The model takes the form of a piecewise smooth set of ODEs, allowing for an efficient analysis of the dynamics.

**Figure 5.**
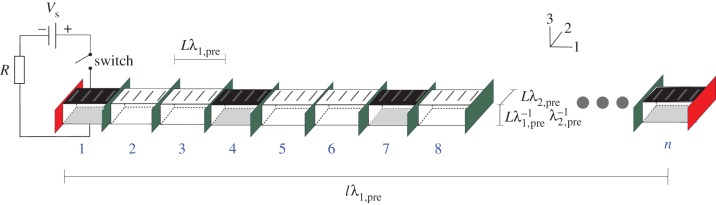
Illustration of the discretization scheme using a prestretched thread. (Online version in colour.)

The DE is discretized using *n* three-dimensional rectangular cuboids, each with mass *m*, which remain rectangular during deformation; this is illustrated schematically in figure 5. We consider elements with natural length *L* in the planar directions, and *T* in the thickness direction. Elements are joined end-to-end in series to form a *thread* of length *l*, with fixed boundary conditions, prestretched in the length direction by a factor *λ*_1,pre_, and in the width direction by the fibre constraints. Elements are represented by two nodes constrained to displace along the thread length only.

As the configuration of each element is determined by the elastic and viscous stretches in the length direction, defined as *λ*_1_ and *ξ*_1_ in the previous section, we now relabel with subscripts denoting the element number. Thus, element *i* has stretch ratio *λ*_i_ with viscous stretch *ξ*_*i*_, both in the length direction. Electroded elements, with potential *V_i_*, are connected to individual RC circuits each with source voltage *V*_S_ and resistance *R*. We define *S_j_* to represent the set of elements that are members of actuator *j*. Applying basic laws and geometry, *V_i_* evolves according to3.5
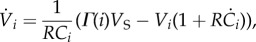
where
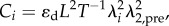
and *Γ*(*i*) represents the global state
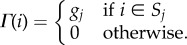


The stress *σ*_*i*_ of an element in the thread length direction is calculated using (3.3)
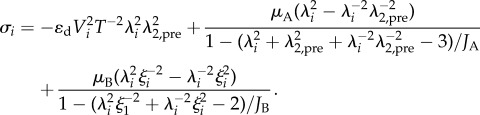


The force on node *i*, *F_i_*, is calculated using the stresses along the thread length in the adjacent elements *i* and *i* + 1 yielding 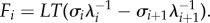 The evolution of the stretch ratios is then defined by the ODEs3.6
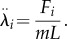


The rate of change of viscous stretch of element *i* is calculated using (3.4) as3.7
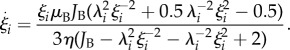


Equations (3.5)–(3.7) form the equations of motion that define the system states *V_i_*, *ξ*_*i*_ and *λ*_*i*_, together with the boundary conditions which constrain the first and last nodes to be fixed.

An efficient way to solve for steady states is to assume activated electroded material to have a uniform stretch ratio, *λ*_A_, implying all passive material will have stretch ratio *λ*_B_. Let 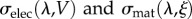 represent the electrical stress and material stress, respectively, of a section of material with stretch *λ* and viscous stretch *ξ* in direction 1 and potential *V*. If there are *a* active blocks and *b* = *l*/*L* − *a* passive blocks, the fixed boundary conditions give an equation for *λ*_B_,3.8
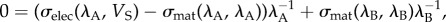
where
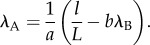


Equation (3.8) can be solved easily using conventional numerical techniques, followed by a consistency check for the global state.

## 4. Pattern creation

We now use the modelling framework developed above to design and analyse controllable systems that are able to switch between different pattern types. We optimize parameters to accentuate the differences in pattern types, maximizing the change in area covered by cells in the actuated and unactuated configurations.

We analyse three pattern types, designed to mimic those seen in biological systems, summarized in table 2. For example, the type II pattern is capable of mimicking the passing cloud display of the cuttlefish, by allowing bands of actuation to propagate along the thread. Additionally, the type III pattern is designed to mimic signalling patterns seen in cephalopods, by choosing rules that result in complex activation sequences to propagate along the thread. Each pattern type is defined with a unique source and rule base.

**Table 2.** Summary of considered patterns and their rule bases.

pattern	description	source *s* definition	activation rule	deactivation rule
type I	sawtooth-like oscillation	mean strain across the agent's cell	***s*** < *r*_on_	***s*** > *r*_off_
type II	unidirectional propagation	mean strain across the cell on one side of the agent	***s*** > *r*_on_	***s*** < *r*_off_
type III	chaotic transitions and striped patterns	mean strain across the cells either side of the agent; half the mean strain if there is one neighbouring cell	*r*_on,1_ < ***s*** < *r*_on,2_	***s*** < *r*_off,1_ OR *r*_off,2_ < ***s***

### 4.1. Optimization of actuator size and spacing

**Figure 6.**
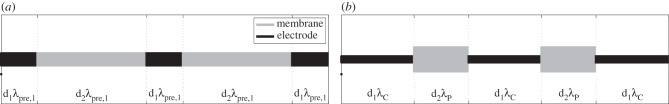
Cross-sectional view (not to scale) of a system using *M* = 3 active cells with the optimal parameters in table 3 in the (*a*) unactuated and (*b*) actuated configurations.

Considering applications such as camouflage and signalling, it is beneficial to maximize the capability to display differences in perceived colour. This is achieved by considering the parameters that maximize the change in length covered by *M* cells, between when all cells are in actuated and unactuated states, illustrated in figure 6 for a particular combination of electroded and non-electroded cells.

In the length direction of the actuated configuration, let cells with stretch ratio *λ*_C_ and natural length *d*_C_ be separated by sections of passive membrane with stretch ratio *λ*_P_ and natural length *d*_P_. The relative change in length covered by the cells is thus given by
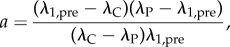
hence the optimal prestretch (which maximizes *a*) is given by
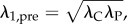
and inter-element spacing
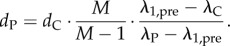


Setting *λ*_C_ and *λ*_P_ to maximize the change is dangerous, since this may put the material close to material strength failure. It is therefore beneficial to leave linear strain margins for cells to actuate into. The buckling margin need not be as high as the material strength failure margin since the steady-state stretch in passive sections is minimized when all cells are fully actuated.

This study considers fabricating active cells that are 50 mm long at prestretch, as this has already been shown to be a plausible order of fabrication size when testing [3], although the system is scalable to other orders of magnitude. The maximal change in area that can be achieved is 42.02%, occurring when *λ*_C_ = 6 and *λ*_P_ = 1. However, to allow a margin against failure modes, the values *λ*_C_ = 5 and *λ*_P_ = 1.25 are used in this paper, which provide an area change of 33.33%. To assess whether this change in area is significant for a particular application, one would need to perform more detailed tests on patterns produced by a real membrane.

The voltage needed to drive the actuator is calculated using (3.8), which gives *V_s_* = 5600 V. This maximizes the localized steady-state area change when all cells are actuated, as fewer actuated cells reduce the stress in the passive membrane. Finally, we set the RC circuits to have a resistance of 10 MΩ, which results in fairly fast charge and discharge times.

The full list of parameters used throughout the rest of this paper is given in table 3. Note that the patterns generated are not unique to this set of parameters but would result in either reduced relative area change or lower failure tolerances.

**Table 3.** System parameters used in the model.

parameter	value (unit)
*λ*_1,pre_	2.5
*λ*_2,pre_	1
*d*_C_	20 (mm)
*d*_P_	40(*M*/(*M* − 1)) (mm)
natural thickness, *T*	0.5 (mm)
density, *ρ*	960 (kg m^−3^)
*R*	10 (MΩ)
*V*_S_	5600 (V)

### 4.2. Type I pattern: sawtooth-like oscillation

**Figure 7.**
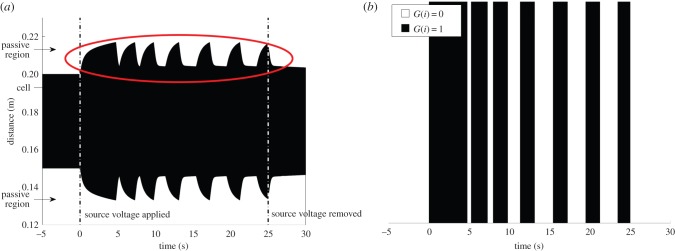
Type I pattern: ‘sawtooth’ oscillatory behaviour, shown circled above, using thresholds of *r*_on_ = 2.9, *r*_off_ = 4.2, a cell of natural length 20 mm in the centre of a thread of natural length 50 mm. Source voltage is applied at *t* = 0 s, and deactivated after 25 s, indicated by dashed lines. (*a*) View of thread, (*b*) global state transition diagram. (Online version in colour.)

The type I rule base and source was configured to enable cells to enter a sawtooth-like oscillation, as shown in figure 7, a shape that reflects the hyperelastic stress response of DE. Such patterns could be useful for signalling applications.

We illustrate the dynamics associated with this rule base using the simplest combination of cells: a single self-sensing cell, in the middle of a longer (passive) thread. To ensure oscillatory behaviour, limits on *r*_on_ and *r*_off_ are chosen to be within the steady-state source of the cell in global states *G* = 1 and *G* = 0, respectively, requiring *λ*_l,pre_ < *r*_on_ < *r*_off_ < *λ*_C_. Greater differences in the thresholds increase the amplitude of oscillation, as the source oscillates (approximately) between them.

Amplitude parameters of *r*_on_ = 2.9, *r*_off_ = 4.2 are studied, for which the amplitude of oscillation is approximately *λ*_1,pre_. Applying source voltage to the cell at *t* = 0 (shown in figure 7) clearly triggers a limit cycle that results in significant optical modulation, with a period of approximately 3.7 s. The sawtooth-like shape occurs because the cell takes longer to expand than contract, a consequence of working against material stresses which arise from prestretch. Similarly, these material stresses increase cell contraction speed, causing the frequency of oscillation to be dependent on the amplitude.

During oscillation, the momentum of the material does not significantly force the material beyond the thresholds, implying viscous forces and electrical gradients act quickly to change the deformation direction.

### 4.3. Type II pattern: unidirectional propagation

When a number of self-sensing cells are placed after a single manual cell, the type II source is capable of propagating sequences of activation along the thread length. By inputting activation sequences into the manual cell, patterns such as the ‘passing cloud’ display of the cuttlefish can be mimicked. To achieve this, the corresponding rule base was designed to sense the state of the neighbouring cell.

The limit on the activation strain *r*_on_ needs to be large enough to avoid activating cells in the initial steady state *G* = 0. This limit should also be small enough to detect the activation of the neighbouring cell in the extreme circumstance when the steady-state source is minimized in state *G* = 1. Together, these imply *λ*_1,pre_ < *r*_on_ < *λ*_C_. Similarly, the limit for *r*_off_ must be larger than the maximal steady-state source when no cells are actuated, to ensure deactivation is detected. Additionally, *r*_off_ is set below *r*_on_ to avoid local oscillations. Therefore, the necessary rule base conditions for propagation are
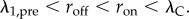


To illustrate propagation, we consider a system with one manual cell (cell 1), followed by 29 self-sensing cells (cells 2–30), and a near-optimal inter-element spacing of natural length *d*_2_ = 40 mm. As cell contraction is faster than actuation, the rule base parameters *r*_on_ = 3.7 and *r*_off_ = 2.6 were chosen because of their fairly even activation and deactivation propagation speeds. The manual cell is set to activate and deactivate every 1 s, ceasing after 8 s, to create regular activation and deactivation propagations.

**Figure 8.**
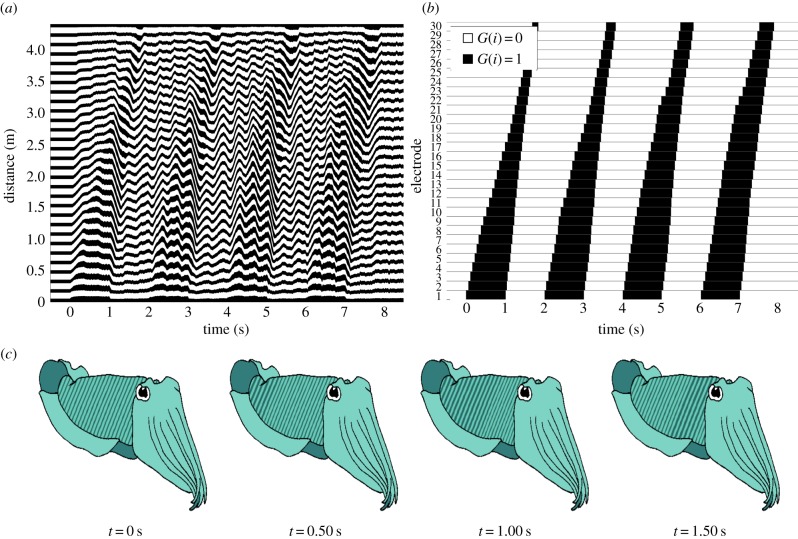
Full propagation of actuation along the thread using parameters *r*_on_ = 3.7, and *r*_off_ = 2.6. Cell 1 (manual cell) activates and deactivates in cycles of 1 s from *t* = 0 for 8 s. (*a*) View of material and (*b*) global state transition diagram; (*c*) the actuation propagation, visualized on an illustration of a cuttlefish body. See the electronic supplementary material for an animation of the simulation. (Online version in colour.)

Figure 8 illustrates regular propagations of activation along the full length of the thread. As tension in the thread causes activation propagations to travel slower than deactivation propagations, cells further away from the manual cell are activated for a shorter length of time. Therefore, the activation time of the manual cell effectively controls the propagation distance of the wave. The approximately regular activation cycles imply that viscous effects do not significantly affect the travelling wave.

The change can be seen more clearly in the global state transition diagram (figure 8*b*), clearly illustrating the width of the travelling actuation waves. The pattern in figure 8 was designed to mimic the passing cloud display [4], where the propagation of actuation is analogous to the bands of colour travelling across the body of a cuttlefish, as shown in figure 8*c*.

### 4.4. Type III pattern: complex behaviour

Our artificial chromatophore skin system is also capable of creating complex, chaotic and long-lasting sequences of actuation, analogous to those produced in some biological systems [4]. We illustrate this by using the type III source. Here, the time evolution of a cell is based on the number of actuated neighbours. If none or two neighbouring cells are actuated, the agent deactivates its cell. If one neighbour is actuated, the agent activates its cell. To implement such behaviour, the corresponding rule base parameters are set to infer the number of actuated neighbours: *r*_off,1_ and *r*_off,2_ detect zero or two actuated neighbours, respectively, and *r*_on,1_ and *r*_on,2_ detects one actuated neighbour. The thresholds should satisfy
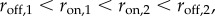
in order to partition ranges for detecting the number of actuated neighbours.

The limits for these rule base parameters are thus determined by analysing the steady-state source as a function of the number of activated cells, using (3.8), for zero, one and two locally activated neighbours. If the ranges of the steady-state source for each neighbour are sufficiently distinct, rule base limits are imposed on the extremes of the ranges to ensure a response to the number of activated neighbours. However, a cell may not respond if its neighbour changes its activation state before the cell approaches the threshold.

**Figure 9.**
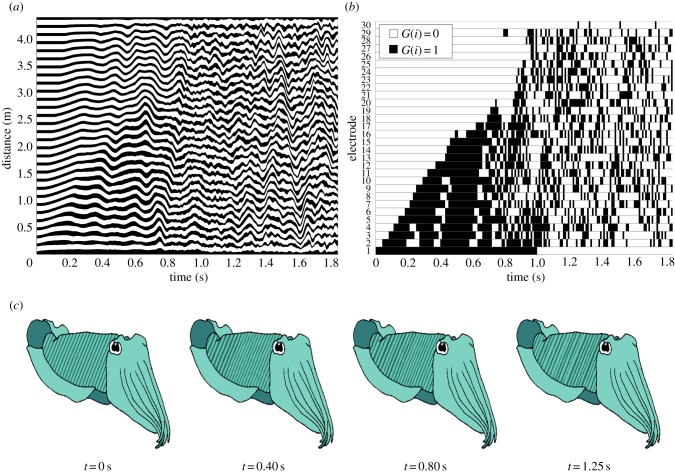
Type III pattern: complex behaviour of the type III source with parameters, *r*_on,1_ = 2.9, *r*_on,2_ = 4.0, *r*_off,1_ = 2.7 and *r*_off,2_ = 4.2, and with the manual cell set to be active for the first 1 s only. The thread starts from rest in state *G* = 0. (*a*) View of thread and (*b*) global state transition diagram. (*c*) Illustrates the complex switching behaviour. See the electronic supplementary material for an animation of the simulation. (Online version in colour.)

An example of a type III pattern is illustrated in figure 9. Here, the manual switch (cell 1) is used to trigger patterns by activating for 0 s ≤ *t* ≤ 1 s. The pattern creates complicated and long-lasting switching behaviours which occur because the switching rule makes it difficult for the thread to reach steady state. In the first 0.6 s, an initial activation propagation similar to type II behaviour is observed as cells respond to an activated neighbour. For *t* > 0.6 s complex behaviour emerges.

## 5. Conclusion

In this paper, we have presented the design of biomimetic dynamic pattern generation systems in soft artificial skin made from DE. By considering linear arrays of DE actuators, we have demonstrated that such systems can exhibit a range of patterned behaviour using simple local rules to control actuation. The basis of our analysis was a viscoelastic continuum model that incorporates the hyper-viscoelastic properties of DEs, optimized to accentuate actuation. Our results suggest that, although pattern generation is not always regular, the system is capable of generating multiple dynamic pattern types analogous to those seen in biological systems, with an expansion ratio of the same order of magnitude as real cephalopods [4]. Patterns include simple sawtooth-like oscillation, unidirectional propagation of actuation sequences mimicking the passing cloud displays in cephalopods, and more complex long-lasting behaviours.

The artificial cephalopod skin system presented here may be extended to applications such as camouflage and signalling. Following the suggestion that some cephalopods communicate using polarized light [25], patterns could be further accentuated by embedding layers of polarizing filters onto the surface of the DE. By strategically varying the direction of the filter along the surface of the thread and layering multiple actuator membranes on top of each other, we could use the dynamics generated here to create complex patterns of light. This would be particularly useful for search and rescue applications, where rescuers need to stand out. To assert the compatibility of pattern propagation speeds for a particular application, further investigation into the accuracy of actuation speeds predicted by the model is required.

Future work will consider adjusting system parameters to improve propagation control and generate new patterns using other local rules, conducting a more extensive analysis of the different pattern types that can be obtained under variation of system parameters, as well as extending the model to simulate patterns in two-dimensional array systems. This is expected to yield further varieties of dynamic patterns, some of which may correspond to those in the natural world.
